# A new approach to improve the hemodynamic assessment of cardiac function independent of respiratory influence

**DOI:** 10.1038/s41598-021-96050-y

**Published:** 2021-08-26

**Authors:** Leslie M. Ogilvie, Brittany A. Edgett, Simon Gray, Sally Al-Mufty, Jason S. Huber, Keith R. Brunt, Jeremy A. Simpson

**Affiliations:** 1 Department of Human Health and Nutritional Sciences, University of Guelph, 50 Stone Road East, Guelph, ON N1G 2W1 Canada; 2 Department of Pharmacology, Dalhousie Medicine New Brunswick, Saint John, NB Canada; 3 Cambridge Electronic Design Limited, Milton, Cambridge, England; 4 IMPART Investigator Team Canada, Saint John, Canada

**Keywords:** Biological techniques, Physiology, Cardiology

## Abstract

Cardiovascular and respiratory systems are anatomically and functionally linked; inspiration produces negative intrathoracic pressures that act on the heart and alter cardiac function. Inspiratory pressures increase with heart failure and can exceed the magnitude of ventricular pressure during diastole. Accordingly, respiratory pressures may be a confounding factor to assessing cardiac function. While the interaction between respiration and the heart is well characterized, the extent to which systolic and diastolic indices are affected by inspiration is unknown. Our objective was to understand how inspiratory pressure affects the hemodynamic assessment of cardiac function. To do this, we developed custom software to assess and separate indices of systolic and diastolic function into inspiratory, early expiratory, and late expiratory phases of respiration. We then compared cardiac parameters during normal breathing and with various respiratory loads. Variations in inspiratory pressure had a small impact on systolic pressure and function. Conversely, diastolic pressure strongly correlated with negative inspiratory pressure. Cardiac pressures were less affected by respiration during expiration; late expiration was the most stable respiratory phase. In conclusion, inspiration is a large confounding influence on diastolic pressure, but minimally affects systolic pressure. Performing cardiac hemodynamic analysis by accounting for respiratory phase yields more accuracy and analytic confidence to the assessment of diastolic function.

## Introduction

Heart failure is the result of structural and functional changes in the myocardium that lead to impairments in ventricular filling and/or ejection of blood^1^. The measurement of ventricular hemodynamics is essential for understanding changes in cardiac physiology that occur in response to experimentally induced pathologies and therapeutic interventions^2^. Direct hemodynamic measurements provide a comprehensive assessment of systolic and diastolic function in experimental models of heart disease. Owing to the tight anatomical and functional relationship between the cardiovascular and respiratory systems, their relationship alters cardiac hemodynamics uniquely. Decreases in intrathoracic pressure during inspiration reduce ventricular pressure and augment venous return^3–5^. Respiratory pressures can even exceed the magnitude of diastolic filling pressure^6,7^. Thus, intrathoracic pressure could independently influence the measures of diastolic and systolic hemodynamics. While respiratory rhythms are known to influence ventricular filling and ejection volumes^3,5,8–11^, the extent to which inspiratory pressures affect the evaluation of diastolic and systolic hemodynamics is unknown.

Intrathoracic pressures oscillate between inspiratory and expiratory phases. Many acute stresses and chronic disease states increase the amplitude of intrathoracic pressure swings throughout respiration. This can occur as a result of obstruction in the airways (e.g., pulmonary edema, inflammation), diaphragmatic weakness, or elevated end-expiratory pressures^6,7,12,13^. These changes in intrathoracic pressure change afterload and venous return^14^, impacting cardiac hemodynamics without changing the intrinsic function of the myocardium per se. Experimental variables that impact respiratory function (e.g., myocardial infarction, pressure-overload heart failure, metabolic impairments)^7,15^ could cause indirect changes in cardiac function that lead to misinterpretation.

Here we aimed to understand how inspiratory pressure influences hemodynamic indices of diastolic and systolic function. To test this, we simultaneously measured left ventricle (LV) pressure and tracheal pressure during eupnea and with increasing severities of respiratory resistance loads to mimic changes in respiratory function expected in animal models of heart failure. Using custom software, diastolic and systolic parameters were then separated into inspiratory, early expiratory, and late expiratory phases of respiration for comparable analysis. We show that diastolic pressure and to a lesser extent, systolic pressure, was predicted by tracheal pressure during inspiration. Conversely, tracheal pressure during late expiration showed no correlation with cardiac pressure, providing an assessment of cardiac function independent of respiratory pressure. Diastolic parameters showed a large percent change from expiratory to inspiratory pressure, while systolic parameters changed minimally. Additionally, respiratory resistance loads increased variability in all cardiac parameters, which was more pronounced in the diastolic parameters. Together our data indicate that respiration is a confounding influence to the evaluation of diastolic function. We show a new approach to remove this influence from hemodynamic analyses to provides a more rigorous assessment of diastolic and systolic function.

## Results

### Respiratory resistance loads alter respiratory function

**Figure 1:**
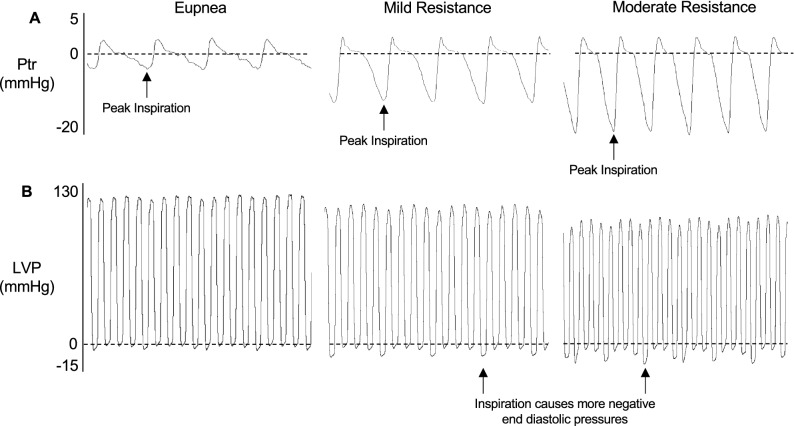
Assessment of tracheal pressure and invasive hemodynamics. Representative tracings of tracheal pressure (Ptr) (**A**) and left ventricle pressure (LVP) (**B**) during eupnea, mild, and moderate resistance loads.

**Figure 2:**
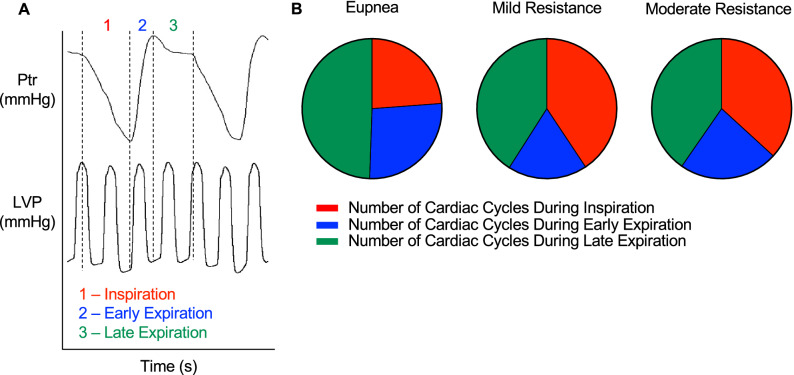
Respiration affects the number of cardiac cycles in each respiratory phase. Representative tracing for a mild respiratory load. Inspiratory (1, red), early expiratory (2, blue), and late expiratory (3, green) phases of respiration were identified by tracheal pressure (Ptr) (**A**). Inspiration was defined between the decline in Ptr and peak negative Ptr. Early expiration was defined between peak negative and maximum Ptr. Late expiration was defined as the plateau phase between maximum Ptr and the fall in Ptr at the start of inspiration. Left ventricle pressure (LVP) was recorded simultaneously with Ptr and parameters of cardiac function were separated by respiratory phase. (**B**) Graphical representation of the number of cardiac cycles that occur during inspiration, early expiration, and late expiration. Data are presented as mean values, n = 7.

We assessed cardiac hemodynamics by invasive catheterization while simultaneously recording tracheal pressure to first understand the influence of resistance loading on respiratory function (Fig. 1). As expected, mild resistance decreased peak inspiratory tracheal pressure compared to eupnea and decreased even further with moderate airway resistance (Table 1). Although not significant (*p* = 0.053), there was a 10% increase in respiratory rate from eupnea with added respiratory loads. Compared to eupnea, resistance loading increased inspiratory time and decreased expiratory time with no difference between the loads themselves. The addition of a mild resistance load decreased early expiratory duration. Moderate resistance loading tended to decrease late expiratory duration compared to eupnea, however this did not reach significance (*p* = 0.066). Respiration can alter cardiac function by either changing the magnitude of intrathoracic pressure or duration of respiratory phase. Thus, we determined the number of cardiac cycles per respiratory phase with increasing respiratory resistance load (Fig. 2A). During eupnea, late expiration had more cardiac cycles compared to inspiration and early expiration, which were equivalent (Fig. 2B, Table 2). Resistance loading altered the number of cardiac cycles that occurred during all respiratory phases. Specifically, mild and moderate resistance similarly increased the number of inspiratory cardiac cycles compared to eupnea by decreasing the number of cycles during expiration. Our data indicate that respiratory loading increased inspiratory time and decreased expiratory time compared to eupnea. Together, these data demonstrate that respiratory loads altered the duration of the respiratory phases, which may enhance phase-specific effects of respiration on cardiac function.

**Table 1:** Respiratory loading increases the work of breathing.

	Eupnea	Mild resistance	Moderate resistance
Ptr_Inspiration_ (mmHg)	− 1.4 ± 0.9	− 13.5 ± 4.1*	− 21.5 ± 4.9*^†^
Frequency (breaths/min)	106 ± 21	116 ± 17	117 ± 12
T_TOT_ (s)	0.585 ± 0.12	0.523 ± 0.07	0.517 ± 0.08
T_I_ (s)	0.184 ± 0.04	0.227 ± 0.03*	0.217 ± 0.03*
T_E_ (s)	0.401 ± 0.10	0.302 ± 0.05*	0.302 ± 0.06*
Early expiratory time (s)	0.134 ± 0.03	0.099 ± 0.02*	0.117 ± 0.02
Late expiratory time (s)	0.267 ± 0.09	0.203 ± 0.04	0.185 ± 0.05

Inspiratory tracheal pressure (Ptr _Inspiration_), frequency, total time of one respiratory cycle (T_TOT_), inspiratory time (T_I_), expiratory time (T_E_), early expiratory, and late expiratory time during eupnea, mild, and moderate resistance loading. Data are presented as mean ± SD.

*p < 0.05 versus eupnea, ^†^*p* < 0.05 mild versus moderate resistance load. All data were analyzed using a within-subject one-way repeated measures ANOVA with Sidak’s correction, n = 7.

**Table 2:** Number of cardiac cycles in each respiratory phase across loading interventions.

	Eupnea	Mild resistance	Moderate resistance
Inspiration	16 ± 4	26 ± 5^d^	27 ± 9^d^
Early expiration	17 ± 6	12 ± 5^b,d^	15 ± 3^b^
Late expiration	32 ± 7^b,c^	26 ± 3^c^	25 ± 4^c^

Data represent the number of cardiac cycles that occurred during each respiratory phase with eupneic breathing and mild and moderate resistance loads. Data are presented as mean ± SD. Two-way within-subjects ANOVA revealed a significant interaction with a main effect of respiratory phase.

^b^Different from inspiration within a respiratory load.

^c^Different from early expiration within a respiratory load.

^d^Different from eupnea within a respiratory phase; *p* < 0.05 for all, n = 7.

### Respiratory phase and resistance loads affect cardiac function

**Figure 3:**
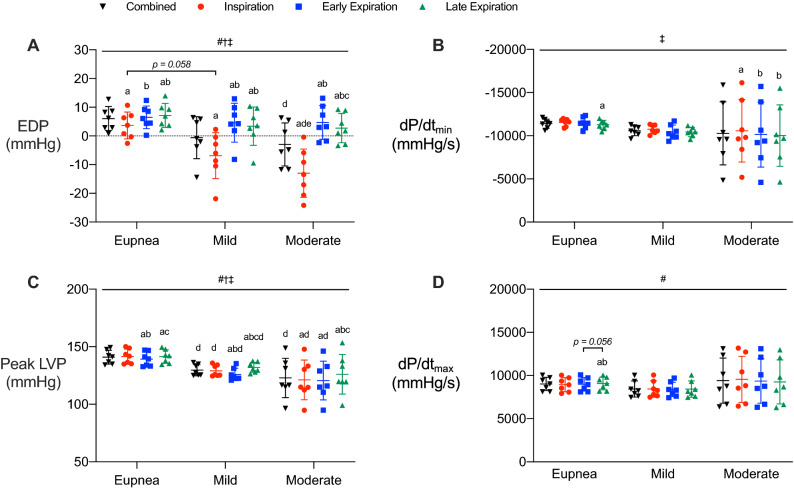
Cardiac function is influenced by respiratory phase and load. Parameters are shown prior to separating by respiratory phase (black; combined) and during inspiratory (red), early expiratory (blue), and late expiratory (green) phases during eupneic breathing and mild and moderate resistance loads. (**A**) End diastolic pressure. (**B**) dP/dt_min_. (**C**) Peak left ventricle pressure. (**D**) dP/dt_max_. Data are presented as mean ± SD. #, significant interaction; †, main effect of respiratory load; ‡, main effect of respiratory phase; a, different from combined within a respiratory load; b, different from inspiration within a respiratory load; c, different from early expiration within a respiratory load; d, different from eupnea within a respiratory phase; e, different from mild resistance within a respiratory phase; *p* < 0.05 for all. All data were analyzed using a within-subject two-way ANOVA with main effects evaluated with one-way repeated measures ANOVAs and Sidak’s correction, n = 7.

To identify how respiratory pressure influences cardiac function, we analyzed systolic and diastolic parameters during inspiratory, early expiratory, and late expiratory phases, and with respiratory phases combined during eupnea and added resistance loads (Fig. 3, S1; https://doi.org/10.6084/m9.figshare.13022759). Diastolic pressures (EDP, Fig. 3A; LVP_min_, Fig. S1D) were higher during early and late expiration compared to inspiration, regardless of respiratory load. The same occurred when comparing EDP and LVP_min_ during early and late expiration to the combined phases, except for EDP at early expiration during eupnea. This indicates that inspiratory-mediated decreases in diastolic pressure drive the observed differences between the combined group and expiratory phases. Interestingly, diastolic pressures were affected by respiratory loading only during inspiration, decreasing as the load increased. This was similar for combined phases due to the negative influence of inspiratory pressure. Diastolic pressures were consistent between early and late expiration across resistance loads, aside from a slight increase during early expiration with moderate resistance. As such, inspiration decreased diastolic pressures, while expiration provided a stable assessment of diastolic pressure, with minimal influence from respiration.

Peak relaxation rate (i.e., dP/dt_min_) was unaffected by respiratory load (Fig. 3B). During eupnea, dP/dt_min_ was lower during late expiration compared to the combined phases (Fig. 3B). The only additional changes observed were with moderate loading, where dP/dt_min_ was higher during inspiration compared to all other respiratory phases and when the phases were combined. Altogether, peak relaxation rate was resistant to influences from respiratory load, with only small changes observed across respiratory phases. The relaxation time constant, Tau (Weiss, Glantz, Logistic), is an index of ventricular relaxation rate. Similar to dP/dt_min_, Tau was not affected by respiratory load (Fig. S1A–C). Thus, relaxation rate was less susceptible to changes in respiratory pressure and phase.

Systolic pressure (i.e., peak LVP) decreased during early expiration compared to all other respiratory phases and when the phases were combined in all respiratory load conditions, except compared to inspiration during moderate resistance (Fig. 3C). Conversely, systolic pressure increased during late expiration compared to all other phases and the combined phases for all loads, excluding during eupnea, where late expiratory and inspiratory systolic pressure were similar. Systolic pressure was affected by resistance load, decreasing with any added load compared to eupnea but with no difference between mild and moderate loads themselves. This occurred for inspiration, early expiration and when the respiratory phases were combined. Late expiratory systolic pressure was the phase least influenced by resistance load, as there was no difference in peak LVP during moderate resistance compared to eupnea or mild load. This is likely because late expiration is the only respiratory phase where intrathoracic pressures are positive throughout.

Respiration had minimal effect on peak contraction rate (i.e., dP/dt_max_); only late expiration was slightly higher than the other phases during eupnea (Fig. 3D). Contraction rate at LVP of 40 mmHg (i.e., dP/dt_@LVP40_) was calculated to provide an afterload-independent index of contractility since dP/dt_max_ is sensitive to preload and afterload. Contractility, independent of afterload, was not different between the respiratory phases during eupnea (Fig. S1E). Mild and moderate respiratory loading for dP/dt_@LVP40_ was higher during early expiration compared to all other phases and when the phases were combined. Conversely, inspiration decreased dP/dt_@LVP40_ during mild and moderate respiratory loads compared to the combined phases and early expiration. These data support a biphasic pattern of contraction rate throughout respiration, where large inspiratory pressures impede contractility and expiratory pressures augment contractility during conditions with additional respiratory load. Surprisingly, although dP/dt_@LVP40_ is less sensitive to changes in ventricle loading than dP/dt_max_, our data show that dP/dt_@LVP40_ is more sensitive to respiratory influences. Lastly, heart rate was also generally unaffected by respiratory function, with only a slight increase during inspiration compared to the combined phases with moderate resistance (Fig. S1F). Altogether, our data indicate that respiratory phases and loads elicited changes in cardiac function that were specific to each systolic and diastolic parameter. Respiration had a greater effect on parameters of cardiac pressure, rather than parameters reflecting contraction or relaxation rate. Of the cardiac parameters most affected by intrathoracic pressure (e.g., EDP, LVP_min_, peak LVP), evaluating data during late expiration minimized aberrations.

### EDP is predicted by tracheal pressure during inspiration, but not during late expiration

**Figure 4:**
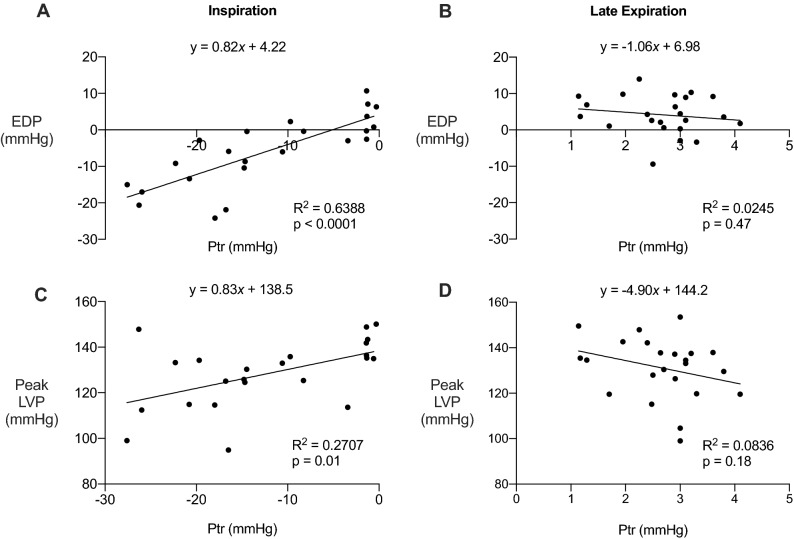
Diastolic and systolic pressures are predicted by inspiratory tracheal pressure. Linear regression of tracheal pressure versus end diastolic pressure (EDP) and peak left ventricle pressure (peak LVP). (**A**) EDP during inspiration. (**B**) EDP during late expiration. (**C**) Peak LVP during inspiration. (**D**) Peak LVP during late expiration. All data were analyzed by linear regression, n = 7.

We performed linear regressions to determine whether respiratory pressure correlates to diastolic and systolic pressure. Analyses were limited to inspiration and late expiration as they have the least and most stable tracheal pressures during respiration, respectively. During inspiration, diastolic pressure decreased as tracheal pressure decreased (Fig. 4A); 64% of the variance observed in EDP was due to changes in tracheal pressure. Conversely, diastolic pressure was unaffected by tracheal pressure during late expiration (Fig. 4B). Systolic pressure also decreased as tracheal pressure decreased during inspiration, with 27% of the variance in peak LVP explained by changes in tracheal pressure (Fig. 4C). During late expiration, systolic pressure was not influenced by tracheal pressure (Fig. 4D). Thus, inspiratory tracheal pressure was a stronger predictor of diastolic compared to systolic pressure, suggesting that diastolic function is more influenced by inspiration than systolic function. In contrast to the large influence of inspiration on cardiac pressure, late expiration had no effect. Therefore, evaluating cardiac function during the late expiratory phase provides a method to assess diastolic and systolic function independent of respiration.

### Respiratory resistance load increases variance in cardiac parameters

**Figure 5:**
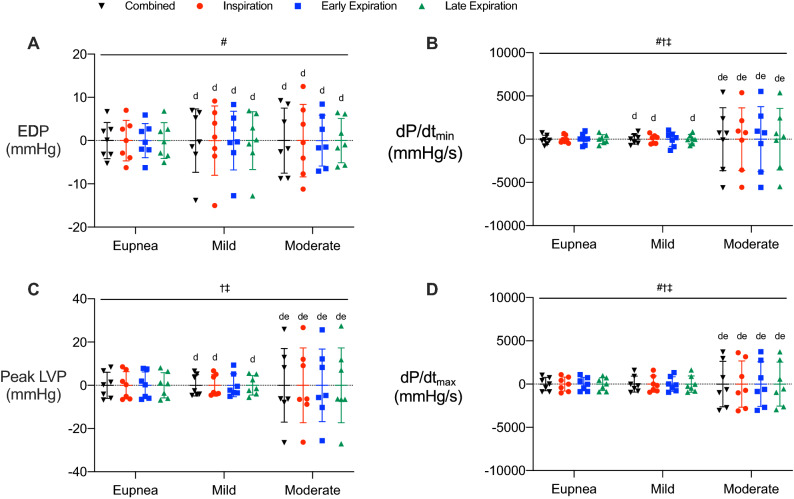
Respiratory resistance loading increases variance in cardiac parameters. Group means were subtracted from individual data points to set all means equal to zero and observe variance in each group. Parameters are shown prior to separating by respiratory phase (black; combined) and during inspiratory (red), early expiratory (blue), and late expiratory (green) phases with eupneic breathing and mild and moderate resistance loads. (**A**) End diastolic pressure. (**B**) dP/dt_min_. (**C**) Peak left ventricle pressure. (**D**) dP/dt_max_. Data are presented as ± SD. #, significant interaction; †, main effect of respiratory load; ‡, main effect of respiratory phase; d, different from eupnea within a respiratory phase; e, different from mild resistance within a respiratory phase; *p* > 0.05 for all. All data were analyzed using a within-subject two-way ANOVA. Where Mauchly’s test of sphericity was significant, one-tailed Pearson’s correlation coefficients were determined. If normality was not assumed, Spearman’s correlations were used, n = 7.

Variability in data collection determines confidence or influences power calculation assumptions that pose ethical and economic impacts on study design. Variability in hemodynamic parameters would increase the demand on sample size. Thus, we determined whether respiratory resistance loads or phases affect variance in diastolic and systolic parameters (Fig. 5, S2; DOI: d10.6084/m9.figshare.13022759; Tables S1–S4; https://doi.org/10.6084/m9.figshare.13022813). With respect to eupnea, both mild and moderate resistance loads proportionately increased variance by a similar amount in diastolic pressures (EDP, Fig. 5A; LVP_min_, Fig. S2D). The addition of a load not the severity of the load itself, impacts variance in diastolic pressure. Load also increased variance in dP/dt_min_ yet, in this parameter the severity of load (not just the addition of it, Fig. 5B) contributed to greater variance. How Tau is measured determines its susceptibility to variance both from an intragroup and intergroup perspective relative to eupnea (Fig. S2). Tau Logistic shows the least variability in any measure, with any load. Tau Weiss has high intragroup variability, which leads to a false expectation of no significant intergroup variance (Fig. S2A). Tau Glantz shows a susceptibility to increased load for elevated intragroup variability and significantly increased intergroup variance (Fig. S2B). The superiority of Tau Logistic is clear, and yet also has significant elevated variance with respect to increasing resistance load, reflecting an impact to accuracy despite superior precision. Respiratory load increases Tau Logistic variance with the severity of load (Fig. S2C). With regard to variance in all diastolic parameters, there was no indication that a specific respiratory phase was driving the variance per se. Tau Logistic is a superior index to evaluate Tau for its precision and—when respiration is accounted for—accuracy over other Tau indices.

With respect to eupnea, resistance loads increased variance in systolic pressure, which was proportional to the severity of load (Fig. 5C). Moderate loading also increased the variance in dP/dt_max_ compared to eupnea or mild load (Fig. 5D). Variance in dP/dt_@LVP40_ was increased with the addition of a resistance load, showing no difference between the loads themselves (Fig. S2E). Moderate loading increased variance in heart rate compared to eupnea (Fig. S2F). While there was little evidence supporting a change in variance during a specific respiratory phase, resistance loading increased variability for all diastolic and systolic parameters.

### Diastolic parameters show greater variance than systolic parameters

To compare variability between diastolic and systolic function, we calculated coefficients of variation for each cardiac parameter across the respiratory phases and loads (Table S5; https://doi.org/10.6084/m9.figshare.13022813). During eupnea and both resistance loads, diastolic parameters (EDP, Tau Weiss, and LVP_min_) had the largest variability of all cardiac parameters measured, regardless of respiratory load. Consistent with the observation of large intragroup variability, Tau Weiss had greater variation coefficients than Tau Glantz or Tau Logistic in most respiratory conditions. This is likely because Tau Weiss does not account for changes in the minimum LV pressure asymptote (e.g., due to intrathoracic pressure changes throughout respiration that vertically shift the ventricular relaxation curve). Conversely, Tau Glantz and Logistic consider these pressure changes when the ventricle is fully relaxed and were thus less affected by respiratory influence (CV < 15% during eupnea). Systolic pressure (i.e., peak LVP) had the smallest variability compared to all other parameters. Similarly, coefficients of variation for most rate parameters (i.e., dP/dt_max_, dP/dt_@LVP40_, dP/dt_min_, and heart rate) were also small (CV < 12%) during eupnea and with mild resistance. Together, these data indicate that variability of diastolic parameters is more sensitive than systolic parameters, regardless of intrathoracic pressure.

### Diastolic pressures have the highest magnitude of change from late expiration to inspiration of all cardiac parameters

**Figure 6:**
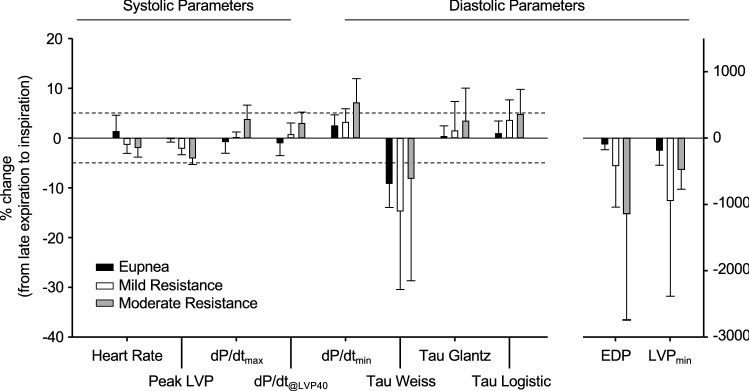
Diastolic parameters are more heavily influenced by inspiration than systolic parameters. Zero identifies the value specific to each parameter at the late expiratory phase of respiration. Vertical bars represent the percentage by which each parameter changes during inspiration from late expiration. Cardiac parameters are shown during eupnea (black), mild resistance (white), and moderate resistance (grey). Dotted horizontal lines are ± 5. Data are presented as mean ± SD, n = 7.

We determined the percent change of systolic and diastolic parameters from late expiration to inspiration (Fig. 6). All systolic parameters showed less than 5% change in all respiratory conditions. Conversely, diastolic pressures (EDP, LVP_min_) had the largest change of all parameters from late expiration to inspiration during eupneic breathing, which was further amplified with resistance loading. This is likely because diastolic pressures are very low compared to systolic pressures (i.e., ~ 5 mmHg during diastole versus ~ 120 mmHg during systole). Therefore, small changes in ventricular pressure (e.g., due to inspiratory influences) have a larger effect on diastolic pressure. Tau Weiss showed a greater percent change compared to Tau Glantz and Tau Logistic. Respiratory function is an important consideration when evaluating diastolic pressure because of the sensitivity of diastolic function to inspiration and the potential for misinterpretations in hemodynamic data when respiratory influences are not considered.

## Discussion

Respiration facilitates a rhythmic influence on cardiac physiology^3,5,16^. Accordingly, cardiac pressures and volumes are altered by changes of intrathoracic pressure throughout respiration. To our knowledge, this is the first study to concurrently measure intrathoracic pressures and cardiac hemodynamics to stratify parameters of systolic and diastolic function by respiratory phase. Additionally, we used respiratory resistance loads to mimic inspiratory pressures expected in experimental models of heart failure, comparing the effects of respiration on cardiac function during normal and resisted breathing. Our salient findings are as follows: 1) inspiratory pressure was a strong predictor of end diastolic pressure and a weak predictor of peak systolic pressure, showing that diastolic function is more sensitive to inspiration than systolic function, 2) cardiac parameters assessed during late expiration were the least affected by changes in tracheal pressure, providing a means to evaluate cardiac function with minimal respiratory influence, 3) respiratory loading increased the variability in all cardiac parameters, which was most pronounced in the diastolic parameters; experimental models with altered respiratory function likely require a greater number of animals to detect group differences, 4) based on our data, researchers should control respiratory rate and depth, and level of anesthesia when evaluating cardiac function, as these alter respiratory function, which in turn influence cardiac function.

**Figure 7:**
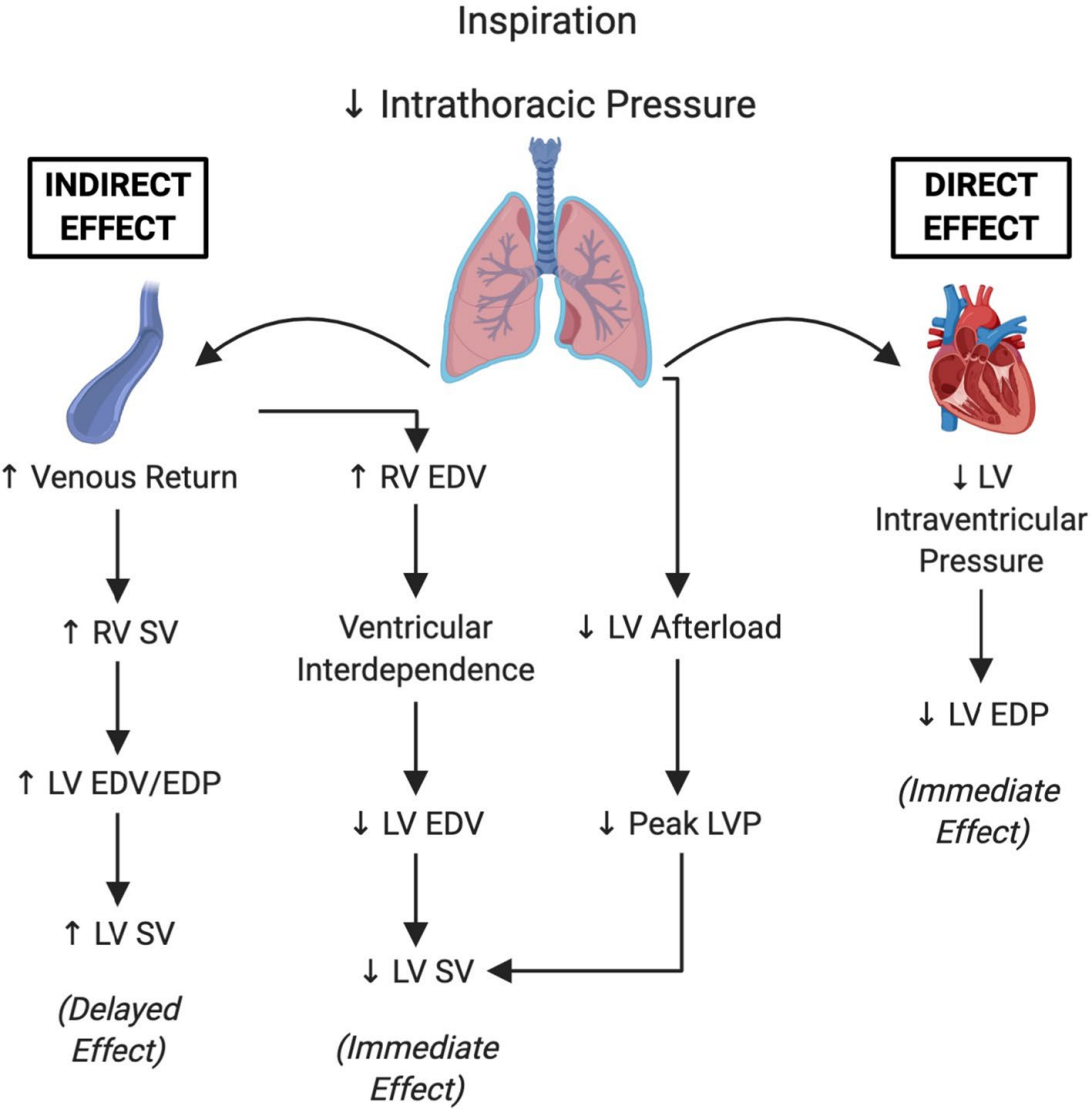
Summary figure. Schematic overview highlighting how inspiratory intrathoracic pressures produce immediate and delayed effects on ventricular pressures and volumes. *RV* Right ventricle, *SV* Stroke volume, *EDV* End diastolic volume, *EDP* End diastolic pressure, *LVP* Left ventricle pressure. Partially adapted from Amoore and Santamore^3^.

Previous literature has shown that respiration produces temporally distinct direct and indirect effects on the heart^3^ (Fig. 7). Decreases in intrathoracic pressure during inspiration reduce ventricular afterload causing a decline in intraventricular pressure. Additionally, the pressure gradient between the thoracic and abdominal cavities during inspiration augments venous return to the right atrium, increasing right ventricle preload and stroke volume. Simultaneously, as right ventricle filling increases, this causes septal shifting—called ventricular interdependence—which transiently decreases left ventricle filling and stroke volume^3,17^. These effects on the left ventricle, secondary to an increase in right ventricle filling, represent the immediate and indirect effects of inspiration. In subsequent cardiac beats, which corresponds with the expiratory phase of respiration, increased right ventricle output increases left ventricle filling and stroke volume^17^; this is the delayed effect of inspiration on left ventricle function. Our data are in agreement with existing literature defining the influence of respiration on cardiac function^3–6,9,11–14,17–20^. We also show that end diastolic pressure, and to a lesser extent, systolic pressure is predicted by inspiratory pressure. As intrathoracic pressure declines during inspiration and is transmitted across the ventricle wall, there is a resulting decrease in intraventricular pressure. Since intraventricular pressure during diastole is a fraction of that during systole, diastolic indices (e.g., EDP, LVP_min_, Tau Weiss) are more sensitive to inspiration than systolic indices. Systolic pressure also decreases during inspiration, however, the effect was minimal compared to diastolic pressure and only observed with an added respiratory load. These findings are critical to our assessment and interpretation of diastolic function with a hemodynamic approach, as alterations in respiratory function may present confounding effects on diastolic pressure without directly changing myocardial function. Interestingly, parameters of cardiac pressure were more influenced by respiratory phase and load than parameters of contraction or relaxation rate. A possible explanation for this observation is that rate parameters exhibited large variability, which may have prevented our ability to detect value differences across the respiratory phases and loads. Alternatively, this finding may suggest that parameters of contraction or relaxation rate are more representative of intrinsic properties of myocardial function (e.g., myofilaments, elastance) and are less affected by external influences, such as intrathoracic pressure.

Research in diastolic dysfunction has surged over the last decade, yet no effective therapies exist to improve myocardial relaxation and/or filling. Elevated end diastolic pressure is recognized as a key contributor to the development of heart failure^21–23^. Indeed, the gold standard for diagnosing heart failure with preserved ejection fraction—characterized by left ventricular diastolic dysfunction with normal systolic function—is elevated end diastolic pressure by cardiac catheterization^24^. Thus, there is a need to comprehensively and accurately evaluate diastolic function to improve our understanding of the pathophysiology of diastolic dysfunction, which could ultimately lead to the development of more effective therapeutics for heart failure by unmasking the definitive functional features with greater precision and accuracy inherent to the fundamental cause. To improve accuracy in hemodynamic data analysis, we suggest reporting diastolic parameters during the late expiratory phase of respiration when respiratory function is not equivalent between experimental groups. This analysis will minimize the confounding effects of respiration on the interpretation of the features of diastolic function.

**Figure 8:**
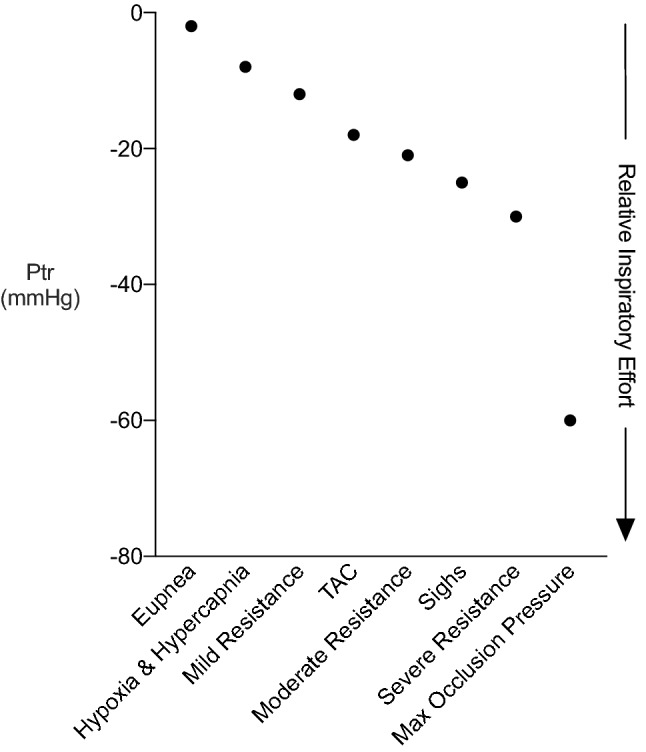
Inspiratory tracheal pressure for various respiratory efforts in rats. Representative tracheal pressure values expected across a range of cardiorespiratory conditions. *Ptr* Tracheal pressure, *TAC* Transverse-aortic constriction to induce pressure-overload heart failure.

In experimental models of heart failure (e.g., pressure-overload heart failure, myocardial infarction, hypoxia) alterations in respiratory function are expected^7,25,26^. Increases or decreases in lung and/or chest wall compliance result in abnormal functional residual capacity (FRC) and intrathoracic pressure values. For example, increases in lung compliance (e.g., emphysema) increase airway resistance, requiring greater respiratory effort. Conversely, decreases in lung compliance (e.g., idiopathic pulmonary fibrosis, pulmonary edema) increase the work of breathing required to inflate a stiff lung. In these pulmonary disease states, intrathoracic pressures are greater and further exacerbate the complication of analysing cardiac function, even in the absence of concomitant cardiovascular disease^27^. We used mild and moderate resistance loads to mimic the inspiratory intrathoracic pressures observed in various cardiac and respiratory pathologies (Fig. 8). Further, agents used to induce and maintain general anesthesia have respiratory depressant effects, which may, in turn, affect cardiac function^28,29^. Alterations in respiratory function, due to anesthetic type/level or experimental intervention, may yield data that does not represent true cardiac function or produces increased intragroup variability. Thus, our findings highlight the importance of taking respiratory rate and depth, and anesthetic level into account when interpreting hemodynamic data.

In the present study, we show that respiratory resistance loads increase variance in all cardiac parameters. Heightened respiratory influences may increase the sample size required to detect differences in hemodynamic function between experimental groups. Although separating cardiac parameters by respiratory phase minimized the influence of inspiration on ventricular pressure, there was no one respiratory phase that showed a consistently reduced variability during eupneic breathing or with respiratory loading. This finding is likely because animals did not all respond the same way to respiratory loading. For example, while some animals showed increased inotropy (contractility) and lusitropy (relaxation rate) with resistance loading, others showed major decreases. These divergent responses greatly increased the variability in cardiac parameters as resistance load increased, hindering our ability to detect subtle changes in variance between the respiratory phases.

The concepts applied in the current analysis may also be valuable for telemetry and echocardiography. Chronic instrumentation (e.g., telemetry) is a growing area of interest, spanning preclinical research and clinical use for investigating cardiovascular health. While not yet scaled for mice, dual pressure transducers are available for larger animals (i.e., rats to humans), providing the opportunity to simultaneously measure cardiac and respiratory pressures. This affords researchers and clinicians the ability to investigate the influence of respiration on cardiac physiology in real time, improving the assessment of diastolic function. Further, for echocardiography, respiratory gating of cardiac function may improve diagnostic sensitivity. The clinical diagnosis of diastolic dysfunction relies on echocardiography, which currently does not take respiratory influence into account. It is exciting that ultrasound machines already provide respiratory waveforms, derived from the ECG. This allows researchers to (retrospectively and prospectively) stratify cardiac function by the various respiratory phases without requiring instrumentation for measures of intrathoracic pressure.

## Study limitations

We performed our analysis with a focus on left ventricle function. The low systolic and diastolic pressures in the right ventricle make it more susceptible to inspiratory influences and changes in afterload than the left ventricle^16^. Future studies should investigate indices of right ventricle function, separated by respiratory phase, to understand how respiration affects the assessment of right ventricle hemodynamics. Secondly, this study was performed in rats, therefore, whether our observations are conserved in mice remains to be investigated. We anticipate that our findings in rats will translate to mouse models since mice also produce large intrathoracic pressure swings throughout respiration^7^. Separating the cardiac parameters into respiratory phases requires LVP and tracheal pressure tracings free from artifacts. Ensuring proper catheter placement is critical (see “Methods” section). This software analysis was created for cardiac and respiratory pressures in rats and is relatively robust in our experience but may need to be optimized in various conditions (e.g., species, strain, age). In disease states or with smaller animals, dynamic variables (e.g., pleural pressure, breathing frequency, residual capacity, and lung/chest wall recoil) exist. We established that respiratory loads greatly affect diastolic parameters in young, healthy animals. Assessing diastolic function by respiratory phase may be of greater importance in conditions of altered cardiac hemodynamics or when the magnitude and/or frequency of respiratory drive increases (e.g., aging, cardiac disease)^7,30,31^. Lastly, this work was performed in male animals only. Future studies could investigate how respiration influences cardiac hemodynamics in females, taking into account the effects of female estrous cycling and hormonal influences on cardiac and respiratory physiology^32,33^. However, despite a small change in size, the structural anatomy is sex consistent and should not be fundamentally different to the conclusions we report here.

## Conclusion

In this study, we provide robust analysis to illustrate the benefit of dividing the respiratory cycle into inspiratory and expiratory phases to assess ventricular systolic and diastolic pressures. Together, our data show that respiration is a confounding influence on the assessment of cardiac hemodynamics. While inspiratory pressures have a slight effect on systolic function, the effect on diastolic function is profound. Given the ongoing interest in characterizing diastolic dysfunction and its contribution to all forms of heart failure, accurate methods for measuring diastolic function are critical. We propose that in scenarios where respiratory function is not equivalent between experimental groups, evaluating hemodynamic parameters during the late expiratory phase of respiration provides the most accurate, respiratory-independent assessment of cardiac physiology. In doing so, we will minimize the confounding influence of inspiration on systolic and diastolic function and limit misinterpretations or failure to identify salient small effects within hemodynamic data that would be pathophysiologically relevant to the mechanism underlying feature changes. This will be important for data reproducibility as research in experimental models of heart failure continues to uncover important insights in disease progression and therapeutic targets for improving cardiac health.

## Methods

### Ethical approval

Experiments, approved by the Animal Care Committee of Queen’s University and in conformity with the Canadian Council on Animal Care guidelines, were conducted on pentobarbital sodium-anesthetized, male Sprague–Dawley rats (300–460 g; 65 mg/kg I.P.), supplemented as required to prevent a pedal reflex. This study was carried out in compliance with the ARRIVE guidelines^34^.

### Assessment of cardiorespiratory function

In brief, after a surgical plane of anesthesia was established, rats (n = 7) were placed supine. Body temperature was maintained at 37.5 °C with a servo-controlled heating pad. One port of a tracheal cannula was connected to a pressure transducer to measure tracheal pressure (used as a surrogate for intrathoracic pressure to identify the beginning and end of each respiratory phase). Inspiration coincides with a decrease in tracheal pressure to its peak negative value and lung volume increases (tidal volume is approximately 7 ml/kg in Sprague–Dawley rats)^35^. During early expiration, tracheal pressure increases from peak negative pressure to its most positive value, then reaches a plateau. During expiration lung volume returns to FRC. For timing of the respiratory phases, we selected tracheal pressure measures over esophageal (a surrogate for pleural pressure) because this provided a cleaner signal, free from movement artifacts (e.g., cardiac contractions), and improved separation of expiration into early and late phases. Esophageal pressure could be used for timing of the respiratory phases, but this presents physiological and technical challenges to obtain a clean signal for automated analysis. The other port of the tracheal cannula was attached to a two-way valve (Hans Rudolf 2300, Kansas City, MO, USA). The right carotid artery was isolated, and a pressure catheter was inserted and advanced into the left ventricle. Once physiological pressures were recorded during eupneic breathing, a small clamp was attached to the inspiratory side of the two-way valve and was fully constricted to produce the maximum inspiratory pressure for each animal. To produce inspirations deeper than eupneic breathing and load the respiratory system, the clamp was partially tightened to generate mild (~ 20–30% of the maximum inspiratory pressure) or moderate (~ 40–50% of the maximum inspiratory pressure) resistance loads. Left ventricle function was recorded at a sampling rate of 1 kHz, then rats were euthanized by pentobarbital overdose. Parameters of left ventricle function (i.e., heart rate; peak left ventricle pressure, peak LVP; end diastolic pressure, EDP; minimum left ventricle pressure, LVP_min_; peak contraction and relaxation rates, dP/dt_max_ and dP/dt_min_, respectively; rate of change of pressure at left ventricle pressure of 40 mmHg, dP/dt_@LVP40_; Tau Weiss; Tau Glantz; Tau Logistic) were analyzed with custom analysis using Spike2 version 10.01 software (CED Spike2, Cambridge, UK) available for download at here (https://ced.co.uk/downloads/scriptspkanal).

Cardiac and respiratory pressure signal distortions, due to improper catheter placement, compromise the precision and accuracy of separating hemodynamic parameters into the various respiratory phases. Real-time monitoring of LVP and dP/dt signals are useful for ensuring proper catheter placement within the LV cavity by the operator. Abnormal catheter placement is observed by extra or distorted peaks in the LVP and/or dP/dt tracings. Further information about proper cardiac catheter placement can be found elsewhere^36,37^. Consistent placement of the tracheal pressure catheter is achieved by using a fixed port on one side of the tracheal cannula. However, mucous secretions can accumulate in the airway, artificially increasing airway resistance, requiring a concomitant increase in respiratory drive. Abnormal or overt increase in respiratory effort or auditory sounds of fluid in the airways (i.e., crackles) is indicative of tracheal secretions. A small catheter connected to a syringe can be used to suction the airway. It is also important that the tracheal cannula does not increase anatomical dead space or airway resistance.

For each respiratory intervention (i.e., eupnea, mild and moderate resistance loads), a 10-s interval of data was obtained from a region of stable left ventricle pressure and parameters were analyzed without separation by respiratory phase (i.e., combined), then separated into inspiratory, early expiratory, and late expiratory phases of respiration for further analysis. In cases where a cardiac cycle fell across two respiratory phases, the cycle was placed into the respiratory phase with the majority (> 50%) of data points for that given cardiac cycle. Tau Weiss, Tau Glantz, and Tau Logistic were calculated as described previously^38^.

### Data analyses

Statistical analyses were conducted with SPSS v. 27 (IBM Corp., Armonk, NY, USA). For each cardiac parameter measured, separate 3 (intervention: eupnea, mild resistance, moderate resistance) × 4 (phase: combined, inspiration, early expiration, late expiration) within-subject analyses of variance (ANOVAs) were conducted. Mauchly’s Test of Sphericity was used to determine whether the variances of the differences between conditions were equal. If significant, the Greenhouse–Geisser correction was used where appropriate. One-way repeated-measures ANOVAs were used to further investigate significant interactions and main effects. A Sidak correction was used for all pairwise comparisons. Linear regression was performed to describe how tracheal pressure affects diastolic and systolic pressure during inspiratory and late expiratory phases. For linear regression, data were analyzed only during inspiratory and late expiratory phases since inspiration generates the largest swings in tracheal pressure and tracheal pressure changes minimally during late expiration. For these tests, statistical significance was determined at *p* < 0.05. The variability of all cardiac parameters was assessed by calculating within-subject coefficients of variation (CV) and, where Mauchly’s Test was significant, one-tailed Pearson’s correlation coefficients were performed. When normality could not be assumed, Spearman’s correlation coefficient was used instead. As these were pair-sampled, they should positively correlate; differences in subject response and thus group variability were determined to be present when *p* > 0.05. Graphs were made using Prism 8 (GraphPad Software Inc., San Diego, CA, USA). All results are expressed as means ± standard deviation (SD) unless otherwise indicated.

## Supplementary Information

Supplementary Figure S1.

Supplementary Figure S2.

Supplementary Legends.

Supplementary Table S1.

Supplementary Table S2.

Supplementary Table S3.

Supplementary Table S4.

Supplementary Table S5.
