# Supplementary figures and images for: A new approach to improve the hemodynamic assessment of cardiac function independent of respiratory influence
Source: Sci Rep. 2021 Aug 26;11:17223. doi: 10.1038/s41598-021-96050-y (PMC8390640; doi:10.1038/s41598-021-96050-y)

▼ Combined    ● Inspiration    ■ Early Expiration    ▲ Late Expiration

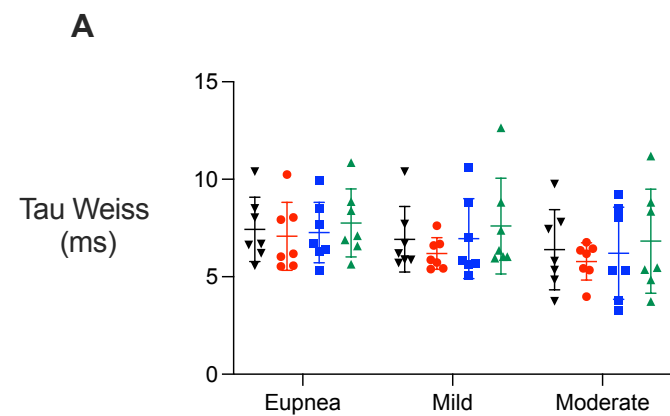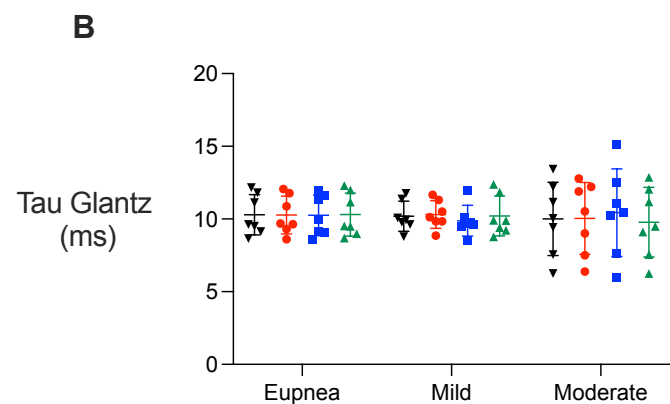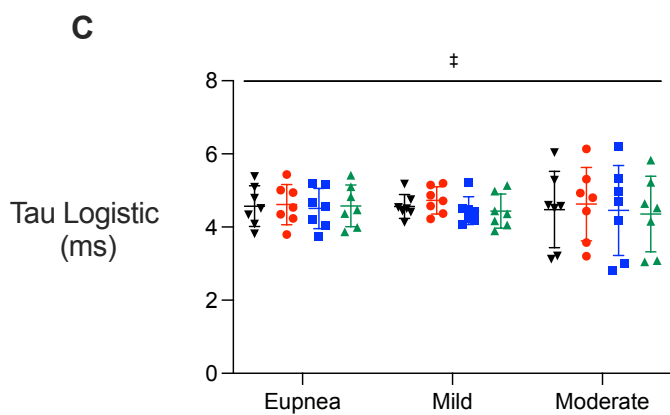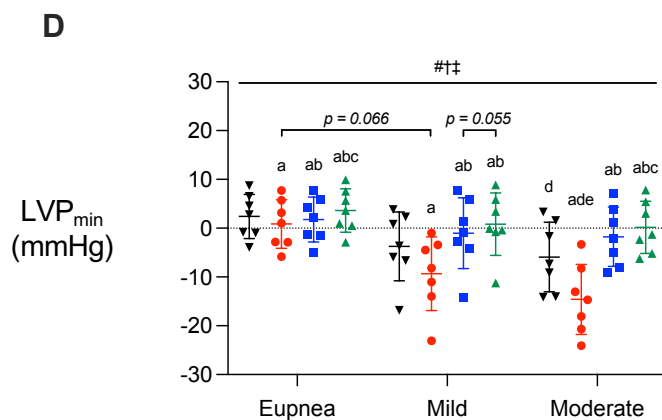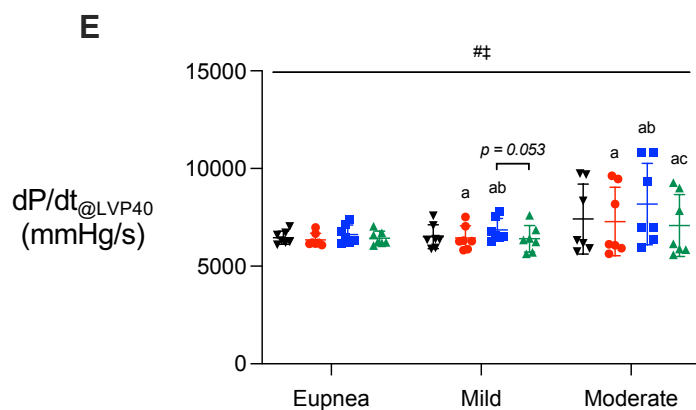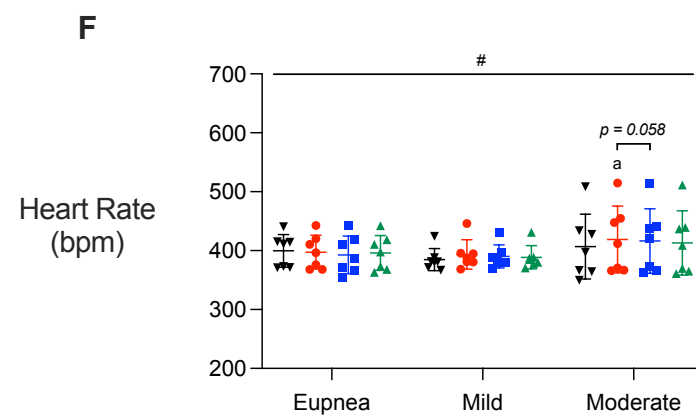

Supplement: Supplementary file 1 — Supplementary Figure S1. [file 41598_2021_96050_MOESM1_ESM.pdf]

▼ Combined    ● Inspiration    ■ Early Expiration    ▲ Late Expiration

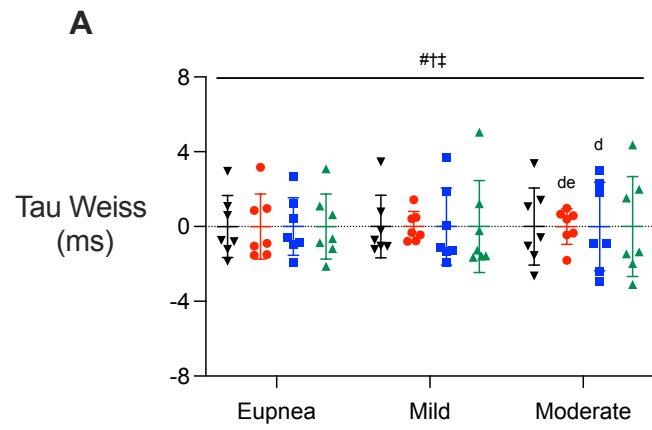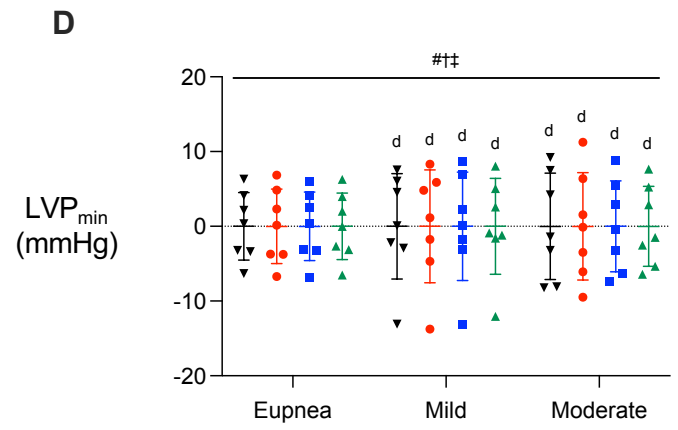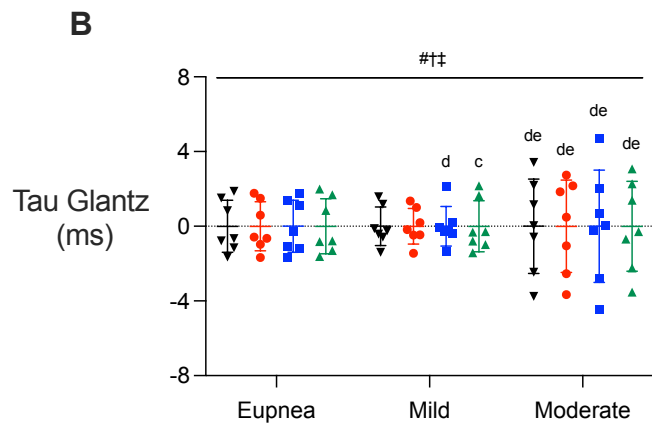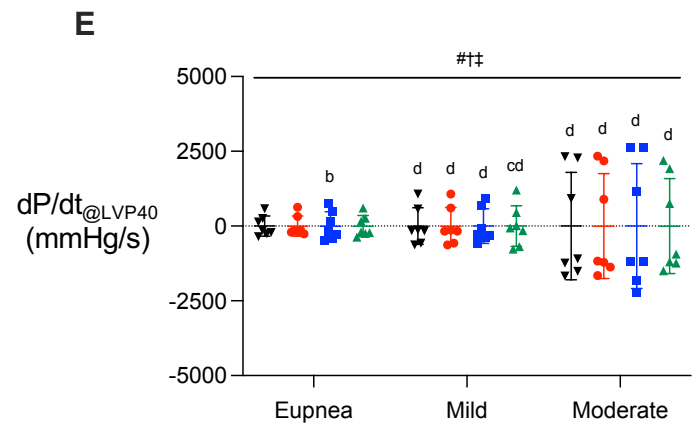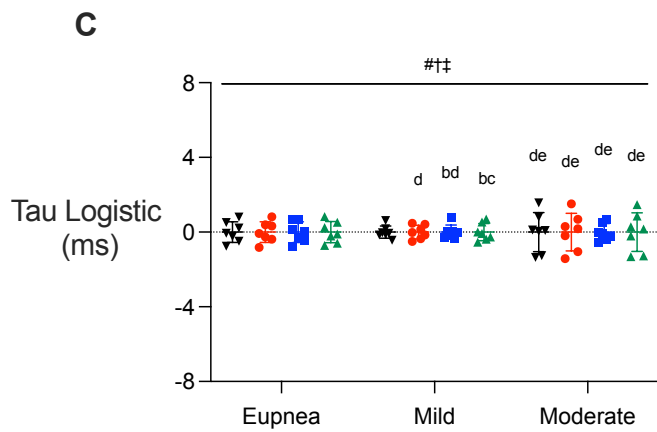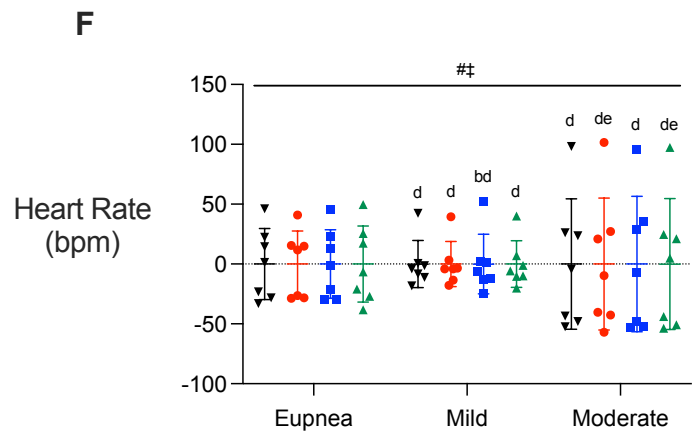

Supplement: Supplementary file 2 — Supplementary Figure S2. [file 41598_2021_96050_MOESM2_ESM.pdf]
