# Supplementary material for: A new approach to improve the hemodynamic assessment of cardiac function independent of respiratory influence
Source: Sci Rep. 2021 Aug 26;11:17223. doi: 10.1038/s41598-021-96050-y (PMC8390640; doi:10.1038/s41598-021-96050-y)
Supplement: Supplementary file 3 — Supplementary Legends. [file 41598_2021_96050_MOESM3_ESM.docx]

**Figure S1. Cardiac parameters are influenced by respiratory phase and load.** Parameters are shown prior to separating by respiratory phase (black; combined) and during inspiratory (red), early expiratory (blue), and late expiratory (green) phases with eupneic breathing and mild and moderate resistance loads. (A) Tau Weiss. (B) Tau Glantz. (C) Tau Logistic. (D) LVP Minimum. (E) dP/dt_@LVP40_. (F) Heart Rate. Data are presented as mean ± SD. #, significant interaction; †, main effect of respiratory load; ‡, main effect of respiratory phase; a, different from combined within a respiratory load; b, different from inspiration within a respiratory load; c, different from early expiration within a respiratory load; d, different from eupnea within a respiratory phase; e, different from mild resistance within a respiratory phase; p < 0.05 for all. All data were analyzed using a within-subject two-way ANOVA with main effects evaluated with one-way repeated measures ANOVAs and Sidak’s correction, n=7.

**Figure S2. Respiratory resistance loading increases variance in cardiac parameters.** Group means were subtracted from individual data points to set all means equal to zero and observe the variance of each group. Parameters are shown prior to separating by respiratory phase (black; combined) and during inspiratory (red), early expiratory (blue), and late expiratory (green) phases with eupneic breathing and mild and moderate resistance loads. (A) Tau Weiss. (B) Tau Glantz. (C) Tau Logistic. (D) LVP Minimum. (E) dP/dt_@LVP40_. (F) Heart Rate. Data are presented as ± SD. #, significant interaction; †, main effect of respiratory load; ‡, main effect of respiratory phase; b, different from inspiration within a respiratory load; c, different from early expiration within a respiratory load; d, different from eupnea within a respiratory phase; e, different from mild resistance within a respiratory phase; p > 0.05 for all. All data were analyzed using a within-subject two-way ANOVA. Where Mauchly’s test of sphericity was significant, one-tailed Pearson’s correlation coefficients were determined. If normality was not assumed, Spearman’s correlations were used, n=7.
