# Supplementary material for: A new approach to improve the hemodynamic assessment of cardiac function independent of respiratory influence
Source: Sci Rep. 2021 Aug 26;11:17223. doi: 10.1038/s41598-021-96050-y (PMC8390640; doi:10.1038/s41598-021-96050-y)
Supplement: Supplementary file 4 — Supplementary Table S1. [file 41598_2021_96050_MOESM4_ESM.pdf]

Table S1. Correlation Coefficients for End Diastolic Pressure.

		**Eupnea**				**Mild Resistance**				**Moderate Resistance**			
		Combined	*Inspiration*	*EE*	*LE*	*Combined*	*Inspiration*	*EE*	*LE*	*Combined*	*Inspiration*	*EE*	*LE*
**Eupnea**	*Combined*	-	0.994<br>P<0.0001	0.976<br>P<0.0001	0.998<br>P<0.0001	0.247<br>P=0.297				0.566<br>P=0.093			
	Inspiration		-	0.980<br>P<0.0001	0.990<br>P<0.0001		0.107<br>P=0.410				0.331<br>P=0.234		
	Early Expiration			-	0.972<br>P<0.0001			0.336<br>P=0.230				0.563<br>P=0.094	
	Late Expiration				-				0.212<br>P=0.324				0.530<br>P=0.111
**Mild Resistance**	*Combined*					-	0.974<br>P<0.0001	0.968<br>P<0.0001	0.989<br>P<0.0001	0.894<br>P=0.003			
	Inspiration						-	0.910<br>P=0.002	0.952<br>P<0.0001		0.940<br>P=0.001		
	Early Expiration							-	0.966<br>P<0.0001			0.828<br>P=0.011	
	Late Expiration								-				0.899<br>P=0.003
**Moderate Resistance**	*Combined*									-	0.919<br>P=0.002	0.993<br>P<0.0001	0.992<br>P<0.0001
	Inspiration										-	0.866<br>P=0.006	0.989<br>P=0.003
	Early Expiration											-	1.000<br>P<0.0001
	Late Expiration												-

EE, early expiration; LE, late expiration. All data were analyzed using a within-subject two-way ANOVA. Where Mauchly's test of sphericity was significant, one-tailed Pearson's correlation coefficients were determined, n=7.
