# Supplementary material for: A new approach to improve the hemodynamic assessment of cardiac function independent of respiratory influence
Source: Sci Rep. 2021 Aug 26;11:17223. doi: 10.1038/s41598-021-96050-y (PMC8390640; doi:10.1038/s41598-021-96050-y)
Supplement: Supplementary file 5 — Supplementary Table S2. [file 41598_2021_96050_MOESM5_ESM.pdf]

Table S2. Pearson's Correlation Coefficients for dP/dt Min.

		Eupnea				Mild Resistance				Moderate Resistance			
		Combined	*Inspiration*	*EE*	*LE*	*Combined*	*Inspiration*	*EE*	*LE*	*Combined*	*Inspiration*	*EE*	*LE*
**Eupnea**	*Combined*	-	0.917 P=0.002	0.987 P<0.0001	0.987 P<0.0001	0.373 P=0.205				-0.047 P=0.460			
	Inspiration		-	0.917 P=0.002	0.875 P=0.005		-0.019 P=0.484				-0.298 P=0.258		
	Early Expiration			-	0.952 P<0.0001			0.697 P=0.041				-0.028 P=0.476	
	Late Expiration				-				0.313 P=0.247				-0.019 P=0.484
**Mild Resistance**	*Combined*					-	0.961 P<0.0001	0.880 P=0.005	0.957 P<0.0001	0.465 P=0.146			
	Inspiration						-	0.826 P=0.006	0.862 P=0.006		0.359 P=0.227		
	Early Expiration							-	0.776 P=0.020			0.509 P=0.122	
	Late Expiration								-				0.487 P=0.134
**Moderate Resistance**	*Combined*									-	0.999 P<0.0001	0.998 P<0.0001	0.999 P<0.0001
	Inspiration										-	0.998 P<0.0001	0.996 P<0.0001
	Early Expiration											-	0.996 P<0.0001
	Late Expiration												-

EE, early expiration; LE, late expiration. All data were analyzed using a within-subject two-way ANOVA. Where Mauchly's test of sphericity was significant, one-tailed Pearson's correlation coefficients were determined, n=7.
