# Supplementary material for: A new approach to improve the hemodynamic assessment of cardiac function independent of respiratory influence
Source: Sci Rep. 2021 Aug 26;11:17223. doi: 10.1038/s41598-021-96050-y (PMC8390640; doi:10.1038/s41598-021-96050-y)
Supplement: Supplementary file 6 — Supplementary Table S3. [file 41598_2021_96050_MOESM6_ESM.pdf]

Table S3. Correlation Coefficients for Peak LVP.

		Eupnea				Mild Resistance				Moderate Resistance			
		Combined	*Inspiration*	*EE*	*LE*	*Combined*	*Inspiration*	*EE*	*LE*	*Combined*	*Inspiration*	*EE*	*LE*
**Eupnea**	*Combined*	-	0.997<br>P<0.0001	0.995<br>P<0.0001	0.999<br>P<0.0001	0.625<br>P=0.067				0.654<br>P=0.055			
	Inspiration		-	0.998<br>P<0.0001	0.993<br>P<0.0001		0.577<br>P=0.088				0.634<br>P=0.063		
	Early Expiration			-	0.990<br>P<0.0001			0.795<br>P=0.016				0.656<br>P=0.055	
	Late Expiration				-				0.659<br>P=0.054				0.648<br>P=0.484
**Mild Resistance**	*Combined*					-	0.995<br>P<0.0001	0.918<br>P=0.002	0.974<br>P<0.0001	0.427<br>P=0.17			
	Inspiration						-	0.918<br>P=0.002	0.950<br>P=0.001		0.344<br>P=0.225		
	Early Expiration							-	0.880<br>P=0.005			0.452<br>P=0.156	
	Late Expiration								-				0.507<br>P=0.123
**Moderate Resistance**	*Combined*									-	0.999<br>P<0.0001	0.997<br>P<0.0001	0.998<br>P<0.0001
	Inspiration										-	0.998<br>P<0.0001	0.997<br>P<0.0001
	Early Expiration											-	0.993<br>P<0.0001
	Late Expiration												-

EE, early expiration; LE, late expiration. All data were analyzed using a within-subject two-way ANOVA. Where Mauchly's test of sphericity was significant, one-tailed Pearson's correlation coefficients were determined. If normality was not assumed, Spearman's correlations (blue) were used, n=7.
