# Supplementary material for: A new approach to improve the hemodynamic assessment of cardiac function independent of respiratory influence
Source: Sci Rep. 2021 Aug 26;11:17223. doi: 10.1038/s41598-021-96050-y (PMC8390640; doi:10.1038/s41598-021-96050-y)
Supplement: Supplementary file 7 — Supplementary Table S4. [file 41598_2021_96050_MOESM7_ESM.pdf]

Table S4. Correlation Coefficients for dP/dt Max.

		Eupnea				Mild Resistance				Moderate Resistance			
		Combined	*Inspiration*	*EE*	*LE*	*Combined*	*Inspiration*	*EE*	*LE*	*Combined*	*Inspiration*	*EE*	*LE*
**Eupnea**	*Combined*	-	0.998<br>P<0.0001	0.999<br>P<0.0001	0.999<br>P<0.0001	0.750<br>P=0.026				-0.117<br>P=-.402			
	Inspiration		-	0.997<br>P<0.0001	0.998<br>P<0.0001		0.736<br>P=0.03				-0.067<br>P=0.443		
	Early Expiration			-	0.997<br>P<0.0001			0.777<br>P=0.02				-0.119<br>P=0.40	
	Late Expiration				-				0.759<br>P=0.024				-0.115<br>P=0.403
**Mild Resistance**	*Combined*					-	0.998<br>P<0.0001	0.996<br>P<0.0001	0.999<br>P<0.0001	0.352<br>P=0.129			
	Inspiration						-	0.993<br>P<0.0001	0.996<br>P<0.0001		0.399<br>P=0.188		
	Early Expiration							-	0.994<br>P<0.0001			0.303<br>P=0.254	
	Late Expiration								-				0.325<br>P=0.238
**Moderate Resistance**	*Combined*									-	0.999<br>P<0.0001	0.999<br>P<0.0001	0.999<br>P<0.0001
	Inspiration										-	0.998<br>P<0.0001	0.997<br>P<0.0001
	Early Expiration											-	0.999<br>P<0.0001
	Late Expiration												-

EE, early expiration; LE, late expiration. All data were analyzed using a within-subject two-way ANOVA. Where Mauchly's test of sphericity was significant, one-tailed Pearson's correlation coefficients were determined, n=7.
