# Supplementary material for: A new approach to improve the hemodynamic assessment of cardiac function independent of respiratory influence
Source: Sci Rep. 2021 Aug 26;11:17223. doi: 10.1038/s41598-021-96050-y (PMC8390640; doi:10.1038/s41598-021-96050-y)
Supplement: Supplementary file 8 — Supplementary Table S5. [file 41598_2021_96050_MOESM8_ESM.pdf]

Table S5. Coefficients of variation for diastolic and systolic parameters.

	Eupnea	*Mild Resistance*	*Moderate Resistance*
**EDP**			
Combined	64%	1120%	234%
Inspiration	118%	108%	60%
Early Expiration	57%	137%	116%
Late Expiration	54%	182%	173%
**dP/dt Min**			
Combined	5%	5%	35%
Inspiration	4%	5%	34%
Early Expiration	6%	8%	37%
Late Expiration	5%	5%	35%
**Tau Weiss**			
Combined	22%	24%	32%
Inspiration	25%	13%	17%
Early Expiration	21%	30%	38%
Late Expiration	22%	32%	39%
**Tau Glantz**			
Combined	14%	10%	25%
Inspiration	13%	9%	25%
Early Expiration	14%	11%	29%
Late Expiration	14%	13%	25%
**Tau Logistic**			
Combined	12%	7%	23%
Inspiration	12%	8%	23%
Early Expiration	12%	9%	28%
Late Expiration	12%	10%	24%
**LVP Minimum**			
Combined	188%	190%	120%
Inspiration	576%	81%	49%

Early Expiration	258%	714%	343%
Late Expiration	123%	773%	3057%
**Peak LVP**			
Combined	4%	4%	14%
Inspiration	4%	4%	14%
Early Expiration	4%	4%	14%
Late Expiration	4%	3%	14%
**dP/dt Max**			
Combined	9%	11%	28%
Inspiration	9%	11%	28%
Early Expiration	9%	10%	27%
Late Expiration	8%	11%	28%
**dP/dt @LVP40**			
Combined	5%	9%	24%
Inspiration	5%	10%	24%
Early Expiration	7%	8%	25%
Late Expiration	5%	11%	22%
**Heart Rate**			
Combined	7%	5%	13%
Inspiration	7%	5%	14%
Early Expiration	7%	6%	13%
Late Expiration	8%	5%	13%

EDP, end diastolic pressure; dP/dt Min, peak relaxation rate; LVP, left ventricle pressure; dP/dt Max, peak contraction rate; dP/dt @LVP40, contraction rate at left ventricle pressure of 40 mmHg, n=7.
